# Neuroprotective effects of punicalagin and/or micronized zeolite clinoptilolite on manganese‐induced Parkinson's disease in a rat model: Involvement of multiple pathways

**DOI:** 10.1111/cns.70008

**Published:** 2024-10-07

**Authors:** Karema Abu‐Elfotuh, Ashwaq N. Abbas, Mazin A. A. Najm, Qutaiba A. Qasim, Ahmed M. E. Hamdan, Amany B. Abdelrehim, Ayah M. H. Gowifel, Aya H. Al‐Najjar, Ahmed M. Atwa, Magy R. Kozman, Azza S. Khalil, Amira M. Negm, Sara Nagdy Mahmoud Mousa, Amira M. Hamdan, Rana H. Abd El‐Rhman, Shaimaa R. Abdelmohsen, Amina M. A. Tolba, Heba Abdelnaser Aboelsoud, Ahmad Salahuddin, Alshaymaa Darwish

**Affiliations:** ^1^ Clinical Pharmacy Department, Faculty of Pharmacy (Girls) Al‐Azhar University Cairo Egypt; ^2^ Al‐Ayen Iraqi University Thi‐Qar Iraq; ^3^ College of Dentistry University of Sulaimanyia Kurdistan Iraq; ^4^ Department of Pharmacy Mazaya University College Thi‐Qar Alnasiriya Iraq; ^5^ Department of Clinical Laboratory Sciences, College of Pharmacy Al‐Ayen Iraqi University Thi‐Qar Iraq; ^6^ Department of Clinical Laboratory Sciences, College of Pharmacy University of Basrah Basrah Iraq; ^7^ Faculty of Pharmacy, Department of Pharmacy Practice University of Tabuk Tabuk Saudi Arabia; ^8^ Biochemistry Department, Faculty of Pharmacy Minia University Minia Egypt; ^9^ Pharmacology and Toxicology Department, Faculty of Pharmacy Modern University for Technology and Information (MTI) Cairo Egypt; ^10^ Pharmacology and Toxicology Department, Faculty of Pharmacy (Girls) Al‐Azhar University Cairo Egypt; ^11^ Pharmacology and Toxicology Department, Faculty of Pharmacy Egyptian Russian University Cairo Egypt; ^12^ Clinical Pharmacy Department, Faculty of Pharmacy Misr University for Science and Technology Giza Egypt; ^13^ Physiology Department, Faculty of Medicine (Girls) Al‐Azhar University Cairo Egypt; ^14^ Oceanography Department, Faculty of Science Alexandria University Alexandria Egypt; ^15^ Department of pharmacology & Toxicology, Faculty of Pharmacy Sinai University – Kantara Branch Ismailia Egypt; ^16^ Anatomy and Embryology Department, Faculty of Medicine (Girls) Al‐Azhar University Cairo Egypt; ^17^ Department of Basic Medical Sciences, College of Medicine Prince Sattam Bin Abdulaziz University Al‐Kharj Saudi Arabia; ^18^ Biochemistry Department, Faculty of Pharmacy Damanhour University Damanhour Egypt; ^19^ Department of Biochemistry, College of Pharmacy Al‐Ayen Iraqi University Thi‐Qar Iraq; ^20^ Biochemistry Department, Faculty of Pharmacy Sohag University Sohag Egypt

**Keywords:** anti‐inflammatory, antioxidant, autophagy, endoplasmic reticulum stress, micronized zeolite clinoptilolite, oxidative stress, Parkinson's disease, punicalagin

## Abstract

**Background:**

Manganism, a central nervous system dysfunction correlated with neurological deficits such as Parkinsonism, is caused by the substantial collection of manganese chloride (MnCl_2_) in the brain.

**Objectives:**

To explore the neuroprotective effects of natural compounds, namely, micronized zeolite clinoptilolite (ZC) and punicalagin (PUN), either individually or in combination, against MnCl_2_‐induced Parkinson's disease (PD).

**Methods:**

Fifty male albino rats were divided into 5 groups (Gps). Gp I was used as the control group, and the remaining animals received MnCl_2_ (Gp II–Gp V). Rats in Gps III and IV were treated with ZC and PUN, respectively. Gp V received both ZC and PUN as previously reported for the solo‐treated plants.

**Results:**

ZC and/or PUN reversed the depletion of monoamines in the brain and decreased acetyl choline esterase activity, which primarily adjusted the animals' behavior and motor coordination. ZC and PUN restored the balance between glutamate/γ‐amino butyric acid content and markedly improved the brain levels of brain‐derived neurotrophic factor and nuclear factor erythroid 2‐related factor 2/heme oxygenase‐1 and decreased glycogen synthase kinase‐3 beta activity. ZC and PUN also inhibited inflammatory and oxidative markers, including nuclear factor kappa‐light‐chain‐enhancer of activated B cells, Toll‐like receptor 4, nucleotide‐binding domain, leucine‐rich‐containing family, pyrin domain‐containing‐3 and caspase‐1. Bcl‐2‐associated X‐protein and B‐cell leukemia/lymphoma 2 protein (Bcl‐2) can significantly modify caspase‐3 expression. ZC and/or PUN ameliorated PD in rats by decreasing the levels of endoplasmic reticulum (ER) stress markers (p‐protein kinase‐like ER kinase (PERK), glucose‐regulated protein 78, and C/EBP homologous protein (CHOP)) and enhancing the levels of an autophagy marker (Beclin‐1).

**Discussion and Conclusion:**

ZC and/or PUN mitigated the progression of PD through their potential neurotrophic, neurogenic, anti‐inflammatory, antioxidant, and anti‐apoptotic activities and by controlling ER stress through modulation of the PERK/CHOP/Bcl‐2 pathway.

## Graphical abstract

ZC: zeolite clinoptilolite, PUN: punicalagin, PD: parkinsonism disease, Mn: manganese, ACHE: acetyl choline esterase, MDA: Malondialdehyde, SOD: Superoxide dismutase, TAC: total antioxidant capacity, DA: Dopamine, NE: norepinephrine, 5HT: serotonin, PCR: polymerase chain reaction, BAX: Bcl‐2‐associated X‐protein, Bcl2: B‐cell leukemia/lymphoma 2 protein, ELISA: Enzyme‐linked Immunosorbent Assay, Enzyme BDNF: Brain‐derived neurotrophic factor, GSK‐3β: Glycogen synthase kinase‐3 beta, glu: glutamate, PGE‐2: Prostaglandin E2, GABA: Gamma‐aminobutyric acid, CHOP: C/EBP Homologous Protein, GRP78: Glucose‐Regulated Protein 78, PERK: protein kinase‐like endoplasmic reticulum kinase, iNOS: Inducible nitric oxide synthase, IL‐1β: Interleukin ‐1β, NF‐kB: nuclear factor kappa‐light‐chain‐enhancer of activated B cells, TLR4: Toll like receptor 4, NLRP3: nucleotide‐binding domain, leucine‐rich–containing family, pyrin domain–containing‐3, COX‐2: Cyclooxygenase 2, TNF‐α: Tumor necrosis factor alpha, Nrf2: nuclear factor erythroid 2‐related factor 2, HO‐1: Heme oxygenase‐1, I.P: Intraperitoneal, P.O: oral.

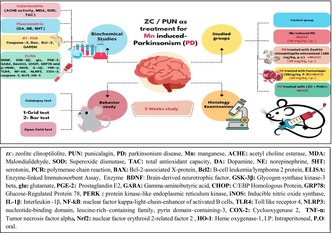

## 1 INTRODUCTION

Manganese is a vital trace element essential for optimal brain function, neurotransmitter synthesis, metabolism, and antioxidant defense mechanisms.^1^ The main valence states of manganese are Mn^2+^, Mn^3+^, and Mn^4+^, with Mn^2+^ ions exhibiting notable stability. Inorganic manganese compounds such as MnCl_2_, MnSO_4_, and MnO_2_ are common in the environment and workplace. Both MnSO_4_ and MnCl_2_ dissolve readily in water and undergo rapid dissociation in aqueous solutions. Previous research has highlighted the need for caution in the use of MnCl_2_ due to the rapid tissue turnover of manganese compared to that of oxide and sulfate salts. These compounds can cause neurological issues in animals and humans, especially with acute exposure to high doses, and their solubility influences their bioavailability and targeting of toxicity, especially in the brain.^2^, ^3^, ^4^ Its overaccumulation in specific brain regions can lead to significant dysfunction in the central nervous system (CNS), identified as manganism. This intriguing condition manifests as a combination of motor dysfunction and concurrent neuropsychiatric and cognitive impairments, resembling the features observed in Parkinson's disease (PD).^5^, ^6^ Therefore, MnCl_2_ is employed as a model for studying parkinsonism.^6^, ^7^, ^8^, ^9^, ^10^, ^11^

Exposure to MnCl_2_ elicits neurotoxicity through a complex interplay of diverse mechanisms, perturbation of neurotransmitter production and metabolism, oxidative stress, neuroinflammation, endoplasmic reticulum (ER) stress, and apoptosis.^12^, ^13^ The accumulation of MnCl_2_ in the globus pallidus of the basal ganglia can lead to dysregulation and misconnection of basal ganglia neurotransmitters, including glutamate (Glu) and dopamine (DA),^14^, ^15^, ^16^ resulting in a decrease in the brain content of the DA^15^ and eventually motor dysfunction and akinesia.^17^, ^18^ An imbalance in neurotransmitters due to manganese exposure can cause mitochondrial malfunction and trigger oxidative stress–inflammation–apoptosis pathways in affected brain areas.^18^, ^19^ In the context of oxidative stress‐induced dysregulation, two significant pathways emerge: the nuclear factor erythroid 2‐related factor 2 (Nrf2) pathway, which governs antioxidant defense genes, including heme‐oxygenase‐1 (HO‐1), and the Toll‐like receptor 4 (TLR4)/nuclear factor kappa‐light‐chain‐enhancer of activated B cells (NF‐kB) pathway, which regulates inflammatory responses and cytokine production.^12^, ^20^ To halt this detrimental cycle, it is imperative to formulate medications capable of addressing oxidative stress, rejuvenating cellular metabolism energy, and/or possessing antioxidative and neuroprotective attributes. Notably, activation of the Nrf2 pathway and modulation of TLR4/NF‐kB have substantial potential as neuroprotective agents against oxidative stress, neuronal injury, aging, and diverse brain diseases.^21^, ^22^, ^23^

ER stress is also involved in the neurodegenerative process in PD.^24^ ER stress is prompted by the accumulation of misfolded or unfolded proteins coupled with hypoxia, leading to the activation of the unfolded protein response (UPR), which functions as a protective mechanism to maintain ER homeostasis.^25^, ^26^ During the UPR, the ER luminal chaperone glucose‐regulated protein 78 (GRP78) is released and binds to three major ER membrane sensors, including protein kinase RNA‐like ER kinase (PERK), which initiates apoptotic signaling pathways, including the proapoptotic C/EBP‐homologous protein (CHOP) pathway.^27^, ^28^ In addition to its role in apoptotic signaling pathways, CHOP has also been identified as a direct regulator of various genes involved in promoting cell survival.^29^

Furthermore, recent research has highlighted the critical role of autophagy dysfunction in the pathogenesis of neurodegenerative conditions.^30^ Beclin1, an essential protein for initiating autophagic proteins that attach to the autophagosome prestructure, is a key player in this process.^31^ Previous studies have suggested that enhancing autophagy may be protective against PD. Thus, it is crucial to investigate the potential benefits of autophagy stimulation and ER targeting as therapeutic strategies for PD.^32^, ^33^

Although medications can greatly improve motor function in Parkinsonism patients, they may also introduce challenging side effects, especially with disease progression. Hence, the pursuit of novel treatments or therapeutic strategies continues, with a focus on addressing the fundamental mechanisms driving Parkinson's disease progression.^23^, ^34^ This includes exploring plant‐derived phytochemicals and nutraceuticals with antioxidant and anti‐inflammatory properties while minimizing toxic effects.^23^, ^34^ Punicalagin (PUN), a prevalent ellagitannin in pomegranate fruit, has drawn attention for its various health benefits.^35^, ^36^ PUN possesses a therapeutic perspective in the management of several neurological disorders, including Alzheimer's disease (AD).^37^, ^38^ Researchers attributed its therapeutic effects to its ability to suppress oxidative stress, inflammatory cascades, and apoptosis.^39^, ^40^ Notably, these findings highlighted the potential of PUN as a promising therapeutic agent for the management of various diseases, such as PD.

Zeolite clinoptilolite (ZC) is a naturally occurring hydrated crystalline alumina silicate with a highly porous structure^41^ that enables it to trap ions and molecules,^42^ including heavy metals such as Mn,^43^ making it a valuable material for industrial and biomedical applications.^44^ In addition to its detoxifying properties, ZC has potent anti‐inflammatory, immune‐boosting, anticarcinogenic, and antioxidative effects^45^, ^46^ and has even been found to positively influence the behavior of rats under environmental stress when administered as a dietary supplement.^47^ Interestingly, despite its widespread use and potential therapeutic applications, the precise molecular and neurological mechanisms underlying its effects remain unclear.^41^ Recent studies suggest that ZC may hold promise as a protective agent against AD, as it has been found to attenuate Aβ aggregation, oxidative stress, and the expression of inflammatory mediators.^47^ In summary, these findings suggested that ZC can serve as a promising therapeutic agent to combat neurodegenerative diseases. ZC cannot cross the blood–brain barrier or the intestinal barrier because of its large particle size. Therefore, we employed micronized ZC instead to improve its kinetics.^41^ Hence, both PUN and micronized ZC hold great promise as innovative and effective therapies for mitigating the symptoms of PD.

Therefore, the present study aimed to investigate the neuroprotective properties of PUN and/or ZC in a cutting‐edge rat model of manganese‐induced PD by exploring its intricate molecular pathways, including oxidative stress, cytoprotective Nrf2 pathways, TLR4/NF‐kβ, ER stress, autophagy, and apoptosis pathways, and the potential synergistic effects they may have.

## 2 MATERIALS AND METHODS

### 2.1 Material

#### 2.1.1 Drugs and chemicals

Sigma–Aldrich Chemical Co., St. Louis, Missouri, USA provided Mn as manganese (II) chloride tetrahydrate (MnCl_2_·4H_2_O) and the micronized ZC. Manganese is present as pink crystals and is freshly dissolved in normal saline.^48^ Clinoptilolite zeolite nanoparticles have the following formula: (Na^+^, K^+^, Ca^2+^, Mg^2+^) ^+6^ [(AlO_2_)^6^ (SiO_2_)^30^]^−6^.24H_2_O.

Punicalagin was purchased from Sigma Chemical Co. (St. Louis, MO, USA) and was dissolved in a normal saline solution.^49^ All reagents and chemicals employed in this investigation were of analytical grade.

#### 2.1.2 Animals

Nile Co., Pharmaceuticals and Chemical Industries, Cairo, Egypt, provided adult male albino rats (weighing 300–320 g). The animals were placed in conventional, controlled environments with unrestricted access to water and a pelleted diet of standard rat chow (12‐h dark/light cycle, 60%–70% humidity, and a temperature of 22+ 2°C). After eliminating water and food from the home cages, the investigators tested the situation 1 h prior to each test for adaptation. All experimental procedures were permitted and supervised by the Animal Care and Use Committee of the Faculty of Pharmacy, Al‐Azhar University, with ethical approval number 219/2021. The handling of animals followed the guidelines outlined in the “Guide for Care and Use of Laboratory Animals,” published by the National Institutes of Health (NIH Publications No. 8023, revised 1978).

### 2.2 Methods

#### 2.2.1 Experimental design

Eighty adult male albino rats (10 weeks of age) were divided into eight groups (*n* = 10/group):

1. The normal control group (NC) served as the normal healthy group.
2. The control treated ZC micronized (100 mg/kg, orally using stomach gavage).^50^, ^51^
3. The control was treated with PUN at the dose of (30 mg/kg, orally using stomach gavage).^49^, ^52^
4. The control was treated with a combination of ZC and PUN at doses (100 and 30 mg/kg, respectively via oral rout using stomach gavage).^50^, ^51^
5. Manganese (Mn)‐group, received MnCl_2_ (10 mg/kg, intraperitoneal (i.p.)).^14^, ^15^
6. ZC‐treated group (Mn and ZC): concurrent administration of Mn and ZC at the dose of (100 mg/kg, orally using stomach gavage).^50^, ^51^
7. PUN‐treated group (Mn and PUN): received Mn and PUN at the dose of (30 mg/kg, orally using stomach gavage).^49^, ^52^
8. Combination group (Mn and combination): combined treated with MnCl_2_, ZC at dose of (100 mg/kg, orally by stomach gavage), and PUN at dose of (30 mg/kg, orally via stomach gavage).

All the medications and Mn were freshly administered every day for 5 weeks.

#### 2.2.2 Behavioral assessments

Behavioral assessments were performed 24 h after the last dose. On the first day, the open field test was conducted, followed by the catalepsy grid test on the second day and the bar test on the third day.

##### Open field test (OFT)

Each individual animal was gently placed in the middle of a wooden square box (80 × 80 × 40 cm) with red walls and white smooth polished ground subdivided by black lines into 16 identical squares (4 × 4). Using a stopwatch, the researchers timed the animals' unrestrained exploration of the area for 5 min while a video camera mounted in front of the test box captured their activities. The following behavioral changes will be recorded and evaluated: ambulation frequency (count of squares interred by the rat during a 5‐min period), rearing frequency (count of times the rate is raised to an upright position on its hind limbs with and without support from the four limbs), and latency time (time from positioning the animal in the center of the box until the 1st move).^53^, ^54^

##### Catalepsy test

This test was applied to assess the motor manifestations of parkinsonism, including akinesia, bradykinesia, and rigidity, by putting the rat into an abnormal posture and recording the time elapsed by the animal to rectify this posture. This time is considered a guide for determining the severity of catalepsy.^55^, ^56^ Both the horizontal bar test and the vertical grid test were employed as catalepsy tests.

###### Grid test

Animals were suspended by their 4 paws on vertical stainless steel meshes (the mesh dimensions were 50 × 40 cm, the space between meshes was 0.9× 1.7 cm, with a wooden frame), and the time taken for the first paw movement was measured.

###### Bar test

In this test, we used a bar that was 9 cm parallel to and above the base. We then placed each animal alone on the bar with both forepaws in the half‐rearing posture, and we timed how long it took to remove one or both paws.

#### 2.2.3 Tissue sampling and preparation

Twenty‐four hours after the behavioral tests, ketamine (80 mg/kg, i.p.) was administered to the animals, and the animals were sacrificed.^57^ The brain of each animal was diced and rinsed with saline. For histological analysis, four brains from each group were preserved in 10% neutral buffered formalin for 24 h. The remaining six brains in each group were divided into two parts. The first part was pulverized in separate batches in ice‐cold PBS (pH = 7.4) to produce a 10% homogenate (w/v). Finally, the homogenate was spun for 10 min at 1800 g at 4°C. The supernatant was used to evaluate biochemical parameters. The other part was kept at −80°C for use in real‐time PCR analyses.

#### 2.2.4 Biochemical assessments

##### Biochemical colorimetric assay

A kit purchased from Sigma–Aldrich Co., St. Louis, MO, USA, was used to measure brain acetyl choline esterase (AChE) activity (MAK119). The levels of malonaldehyde (MDA) (Cat No MD 2528), superoxide dismutase (SOD) activity (Cat No SD 2520), and total antioxidant capacity (TAC) (Cat No TA 2512) in brain tissue homogenates were evaluated using commercially available kits purchased from Biodiagnostic, Giza, Egypt, in compliance with the manufacturer's orders.

##### Fluorometric assay

In brief, brain monoamines (DA, NE, and 5‐HT) were promptly measured fluorometrically using the methods outlined by Ciarlone.^58^ Prior to being detected fluorometrically. Monoamines are oxidized to their “adrenochromes” and then converted to their “adrenolutins” before being detected fluorometrically. The adrenolutins for DA, NE, and 5‐HT were estimated fluorometrically at excitation/emission wavelengths of 320–385, 385–485, and 360–470 nm, respectively. Using standard solution fluorescence, the concentrations were determined as nanograms per gram of fresh tissue.

##### Enzyme‐linked immunosorbent assay (ELISA)

Levels of brain‐derived neurotrophic factor (BDNF), glycogen synthase kinase‐3 beta (GSK‐3β), Glu, prostaglandin E2 (PGE‐2), GABA, Beclin1, CHOP, GRP78, p‐PERK, and nucleotide‐binding domain, leucine‐rich‐containing family, and pyrin domain‐containing‐3 (NLRP3) were measured using commercial ELISA kits (#MBS703433, #MBS766198, #MBS756400, #MBS262150 and #MBS269152, #MBS2706719, #MBS1607940, #MBS035991, #MBS2511166 and MBS2033695 My BioSource, San Diego, USA, respectively). Commercial ELISA kits from Sunlong Biotech Co., Zhejiang, China, were used to measure the concentrations of inducible nitric oxide synthase (iNOS), interleukin 1β (IL‐1β), tumor necrosis factor α (TNF‐α), TLR4, NF‐kB, cyclooxygenase 2 (COX‐2), caspase‐1, Nrf2, and HO‐1 in the brain (Cat Nos SL0365Ra, EL0040Ra, SL0722Ra, SL0699Ra, SL1661Ra, SL0218Ra, SL1601Ra, SL0985Ra, and NSL1430r, respectively). Based on the manufacturer's instructions, all these protein biomarkers were detected in the 10% brain tissue homogenate supernatant.

##### Quantitative real‐time polymerase chain reaction (real‐time PCR)

To assess the gene expression of the apoptotic biomarkers Caspase‐3, Bcl‐2‐associated X‐protein (BAX), and B‐cell leukemia/lymphoma 2 protein (Bcl‐2), total RNA was extracted with a Qiagen tissue extraction kit (Qiagen, USA), and 1 μg of total RNA was added to a high‐capacity cDNA reverse transcription kit (Revert Aid Premium Reverse Transcriptase kit, Fermentas, USA). Afterward, the product was amplified with a Maxima SYBR Green qPCR kit (Fermentas, USA) and detected with an ABI Prism 7500 sequence detector system (Applied Biosystems, Foster City, CA). Table 1 shows the sequences of the primers used. The researchers followed the manufacturer's directions for every procedure. The comparative Ct method was used to assess the relative expression levels of the target genes. The results are presented as the fold change in the background in the experimental group after all values were adjusted to GAPDH gene expression.

**TABLE 1:** List of primer sequences for *caspase‐3*, *Bax, Bcl‐2*, and *GAPDH* used for qPCR analysis of rat tissue. Gene forward and reverse primer sequences.

Gene	Gene forward and backward primer sequence	Accession number
*Bax*	F: 5′‐CGGCGAATTGGAGATGAACTGG‐3′ R: 5′‐CTAGCAAAGTAGAAGAGGGCAACC‐3′	NM_031530
*Bcl‐2*	F: 5′‐TGTGGATGACTGACTACCTGAACC‐3′ R: 5′‐CAGCCAGGAGAAATCAAACAGAGG‐3′	NM_199267
*Caspase‐3*	F: 5′‐GTGGAACTGACGATGATATGGC‐3′ R: 5′‐CGCAAAGTGACTGGATGAACC‐3′	NM_012922.2
*GAPDH*	F: 5′‐AACTCCCATTCCTCCACCTT‐3′ R: 5‐GAGGGCCTCTCTCTTGCTCT‐3′	NM_017008.4

Abbreviations: BAX, Bcl‐2‐associated X‐protein; Bcl‐2, B‐cell leukemia/lymphoma 2 protein; Caspase‐3, cysteine‐aspartic acid protease; GAPDH, glyceraldehyde‐3‐phosphate dehydrogenase.

#### 2.2.5 Histopathological examinations

The samples were cleaned and stained for inspection under a light microscope. After being fixed in 10% formalin for 1 day, the brain samples were rinsed with water. For dehydration, alcohol (methyl, ethyl, or absolute ethyl) was serially diluted. For 24 h, the samples were heated to 56°C in a hot air oven, cleaned in xylene, and embedded in paraffin. A Leica Microtome® (#Leica RM2125) was used to prepare paraffin bee wax tissue blocks for sectioning at a thickness of 4 μm.^59^ On glass slides, the resulting tissue sections were reassembled and deparaffinized. After that, the slices were stained with H&E for routine histological inspection using a light electric microscope (Leica® microscope DM500) equipped with an AmScope® microscope digital camera from Leica Biosystems in Buffalo Grove, Illinois, USA, at a magnification power of 40×. Brain tissue lesions were assessed quantitatively based on histopathological changes observed across various brain regions, including the degree of nuclear pyknosis and degeneration, in comparison to those in the normal control group. Scoring was conducted by counting pyknotic and shrunken neurons in 10 microscopic fields using ImageJ 1.53t.^60^

#### 2.2.6 Statistical analysis

The data are expressed as the mean ± SEM and the normal distribution of data was tested using the Kolmogorov–Smirnov test. For parametric data, one‐way ANOVA was performed, followed by Tukey's post‐hoc multiple comparisons test to assess group variation, with a significance level set at *p* < 0.05. For non‐parametric data, the Kruskal–Wallis test was used, followed by Dunn's post‐hoc multiple comparisons test. Statistical analysis and data analysis were performed using GraphPad Prism® (version 5, ISI, USA).

## 3 RESULTS

In this work, three separate normal control groups of rats were subjected to different interventions, ZC, PUN, and their combination. Despite these various interventions, none of the three control groups showed significant differences in any of the measured parameters or histopathological findings compared to the normal control group. Therefore, the results were presented to avoid the complexity of the data (Table S1 and Figure S1).

### 3.1 Effects of ZC or PUN alone or in combination on motor function in the OFT and catalepsy scores in both the bare and grid tests on MnCl_2_ ‐induced PD

After repeated exposure to MnCl_2_, the rats' motor function and coordination significantly deteriorated. In the OFT, ambulation and rearing frequencies were significantly reduced by 84.27% and 78.48%, respectively, and their latencies were significantly increased by 419.2% relative to those of the healthy controls (Table 2). The addition of ZC, PUN, or their combination significantly induced rearing (2.35‐, 3.70‐, and 5.65‐fold, respectively) and ambulation (2.74, 3.37, and 3.6‐fold, respectively), as well as a significant decrease in latency time (76.9%, 74.9%, and 78.8%, respectively). However, the rearing and ambulation frequencies were significantly lower than those in the control by 47.23% and 25.9%, respectively, in ZC and by 19.4% and 5.9%, respectively, in PUN, with significant increases in latency time of 19.7.9% and 29%, respectively, 9%, compared to the negative control group. The combination of ZC with PUN normalized these motor functions (Table 3).

**TABLE 2:** Effect of micronized zeolite clinoptilolite and punicalagin, either alone or in combination, on motor functions in the open field test and on catalepsy scores in both the bar and grid tests in MnCl_2_‐induced PD.

Behavior test	NC	Mn	Mn + ZC	Mn + PUN	Mn + COMB
Open field test					
Rearing frequency (No of rearing/3 min)	21.17 ± 0.601	3.33 ± 0.67^a^	11.17 ± 0.60^b^	15.67 ± 0.67^b^, ^c^	22.17 ± 0.54^b^, ^c^, ^d^
Ambulation frequency (No of squares/3 min)	31 ± 0.58	6.67 ± 0.67^a^	25 ± 0.58^b^	29.17 ± 0.95^b^, ^c^	30.83 ± 0.87^b^, ^c^
Latency time (sec)	1.67 ± 0.33	8.67 ± 0.21^a^	2 ± 0.26^b^	2.17 ± 0.167^b^	1.83 ± 0.31^b^
Catalepsy score (sec)					
Bar test	1.33 ± 0.21	11.67 ± 0.49^a^	3 ± 0.26^b^	2.83 ± 0.31^b^	1.67 + 0.33^b^
Grid test	1.67 ± 0.22	10.42 ± 0.38^a^	2.3 ± 0.28^b^	2.42 ± 0.26^b^	1.83 ± 0.27^b^

*Note*: NC: normal control rats, Mn: MnCl_2_, control group: rats treated daily with MnCl_2_ for 5 weeks to develop PD, Mn + ZC: rats that received MnCl_2_+ zeolite clinoptilolite nanoparticles, Mn + PUN: rats that received MnCl_2_ + punicalagin, Mn + COMB: rats that received a combination of MnCl_2_+ drugs (ZC and PUN). The data are presented as the means ± SEMs, *n* = 6/group; Mn, Mn + PUN. The data are presented as the means ± SEMs, *n* = 6/group. For parametric data, one‐way ANOVA was performed followed by Tukey's multiple comparisons test to assess group variation, with a significance level set at *p* < 0.05, while the Kruskal–Wallis test was used for nonparametric data, followed by Dunn's post hoc multiple comparison tests.

^a^ Significant difference from NC.

^b^ Significant difference from Mn.

^c^ Significant difference from Mn + ZC.

^d^ Significant difference from Mn + PUN; *p‐*value < 0.05.

**TABLE 3:** Effect of micronized zeolite clinoptilolite and punicalagin, either alone or in combination, on the lesion scores of brain tissue (nuclear pyknosis and neuronal degeneration in the cerebral cortex, fascia dentate and subiculum of the hippocampus, striatum, and substantia nigra, together with plaques in striatal areas) in MnCl_2_‐induced PD.

Area group	Cerebral cortex medians (min–max)	Subiculum medians (min–max)	Fascia dentate medians (min–max)	Striatum medians (min–max)	Substantia nigra medians (min–max)
NC	0 (0–0)	0 (0–0)	0 (0–0)	0 (0–0)	0 (0–0)
Mn	20 (16–23)^a^	19 (17–23)^a^	75 (66–85)^a^	11 (8–13)^a^	11 (9–13)^a^
Mn + ZC	2 (1–4)^b^	1 (0–4)^b^	5 (3–7)^b^	2 (0–4)^b^	4 (2–5)^a^, ^b^
Mn + PUN	1 (0–3)^b^	2 (0–2)^b^	3 (1–5)^b^	2 (0–3)^b^	2 (0–3)^b^
Mn + COMB	1 (0–2)^b^	1 (0–3)^b^	1 (0–2)^b^	1 (0–3)^b^	1 (0–2)^b^

*Note*: NC: normal control rats fed a rodent chow diet; Mn: MnCl_2_, control group: rats subjected daily to MnCl_2_ for 5 weeks to induce PD; Mn + ZC: rats that received MnCl_2_ + zeolite clinoptilolite nanoparticles (ZC); Mn + PUN: rats that received MnCl_2_ + Punicalagin (30 mg/kg P.O.); Mn + COMB: rats that received a combination of MnCl_2_ + drugs (ZC and PUN). Data are presented as medians (min–max) from four rats. Statistical analysis was carried out using the nonparametric Kruskal–Wallis test followed by Dunn's multiple comparison test.

^a^ Significant difference from NC.

^b^ Significant difference from Mn; *p*‐value < 0.05.

Similarly, the catalepsy scores in the bare and grid tests increased markedly after MnCl_2_ administration (8.7‐ and 6.2‐fold, respectively), compared with those in the control group. Compared to those of the PD group, ZC, PUN, and their combination significantly reduced the catalepsy scores on the bare (74.1%, 75.8%, and 86.2%, respectively) and grid tests (77.8%, 76.9%, and 82.6%, respectively).

Therefore, the protective effect of ZC was like that of PUN, which has been proven to have a fortifying effect on Mn‐induced deterioration of working ability, learning skills, and memory. The combination of ZC and PUN had a synergistic effect on protecting against working and learning and memory loss. In addition, ZC, PUN, and their combination can return these functions to normal levels almost equal to those of controls.

### 3.2 Effects of ZC or PUN alone or in combination on brain ACHE activity and neurotransmitter levels in MnCl_2_ ‐induced PD

**FIGURE 1:**
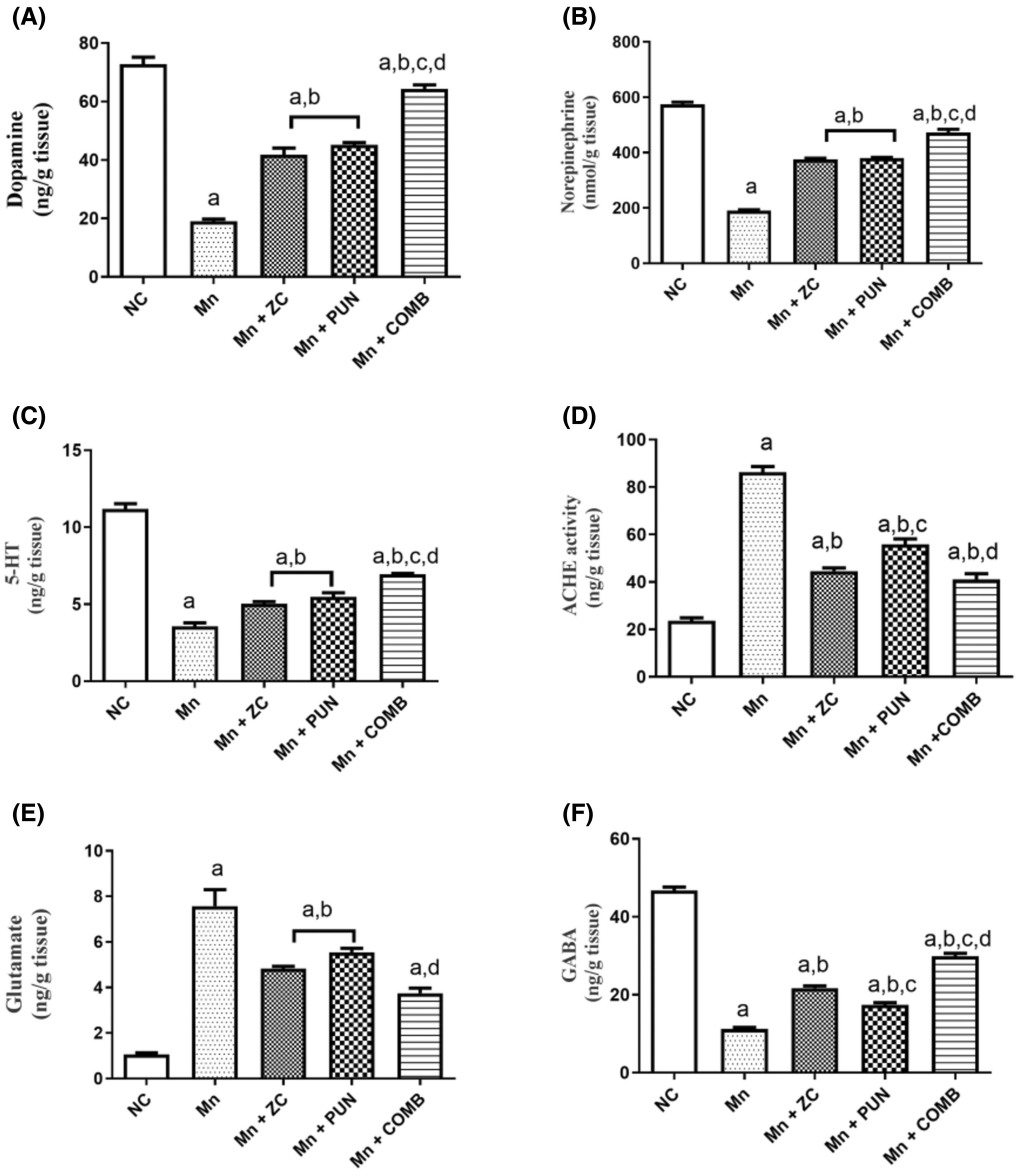
Effects of micronized zeolite clinoptilolite and punicalagin, either alone or in combination, on brain neurotransmitter levels (A) DA, (B) NE, (C) 5‐HT, (D) ACHE activity, (E) glutamate, and (F) GABA in MnCl_2_‐induced PD. NC, Normal control rats fed a rodent chow diet; Mn: MnCl_2_, control group, Rats were treated daily with MnCl_2_ for 5 weeks to develop PD; MnCl_2_ + ZC, Rats received MnCl_2_ + zeolite clinoptilolite nanoparticles; Mn + PUN, Rats received MnCl_2_ + punicalagin; MnCl_2_ + COMB, Rats received a combination of MnCl_2_+ drugs (ZC and PUN); PD, Parkinson's disease; ZC, zeolite clinoptilolite; PUN, Punicalagin; COMB, Combination of ZC and MnCl_2_; 5‐HT, 5‐hydroxytryptamine or serotonin; DA, Dopamine; NE, Norepinephrine; ACHE, Acetyl choline esterase; GABA, Gamma‐aminobutyric acid. The data are presented as the means ± SEMs, *n* = 6/group; ^a^significant difference from NC; ^b^significant difference from Mn; ^c^significant difference from Mn + ZC; ^d^significant difference from Mn + PUN. For parametric data, one‐way ANOVA was performed followed by Tukey's multiple comparisons test to assess group variation, with a significance level set at *p* < 0.05, while the Kruskal–Wallis test was used for nonparametric data, followed by Dunn's post hoc multiple comparison tests.

MnCl_2_ administration significantly decreased the levels of NE, DA, 5‐HT, and GABA in brain tissue (73.9%, 66.8%, 58.1%, and 61.9%, respectively) and increased ACHE activity and glutamate levels (approximately 72.6% and 3.1‐fold, respectively), relative to those in normal animals. However, the supplementation of rats with micronized ZC, PUN, and their combinations considerably increased the levels of these neurotransmitters by approximately 54.4%, 57.8%, and 70.4%, respectively, for DA; 49.3%, 49.9%, and 59.7%, respectively, for NE; 28.0%, 35.0%, and 49.2%, respectively, for 5‐HT; 93.7%, 54.4%, and 166%, respectively, for GABA; and decreased ACHE activity by approximately 48.8%, 35.1%, and 52.4%, respectively, and glutamate levels by 36.0%, 26.6%, and 50.6%, respectively, compared to those in the MnCl_2_‐treated group. The drug combination substantially enhanced brain DA, NE, 5‐HT, and GABA levels relative to monotherapy, while the combination therapy had the greatest effect on lowering ACHE activity and glutamate (Figure 1).

As a result, ZC offered fortifications such as PUN, which has guarded against Mn‐induced dysregulation of catecholamine levels and neurotransmitters. In addition, the combination of ZC and PUN had a synergistic effect on protection against these changes in the levels of all the measured neurotransmitters, which returned to normal levels.

### 3.3 Effects of ZC or PUN alone or in combination on brain oxidative stress, lipid peroxidation, and cytoprotective biomarker levels in MnCl_2_ ‐induced PD

**FIGURE 2:**
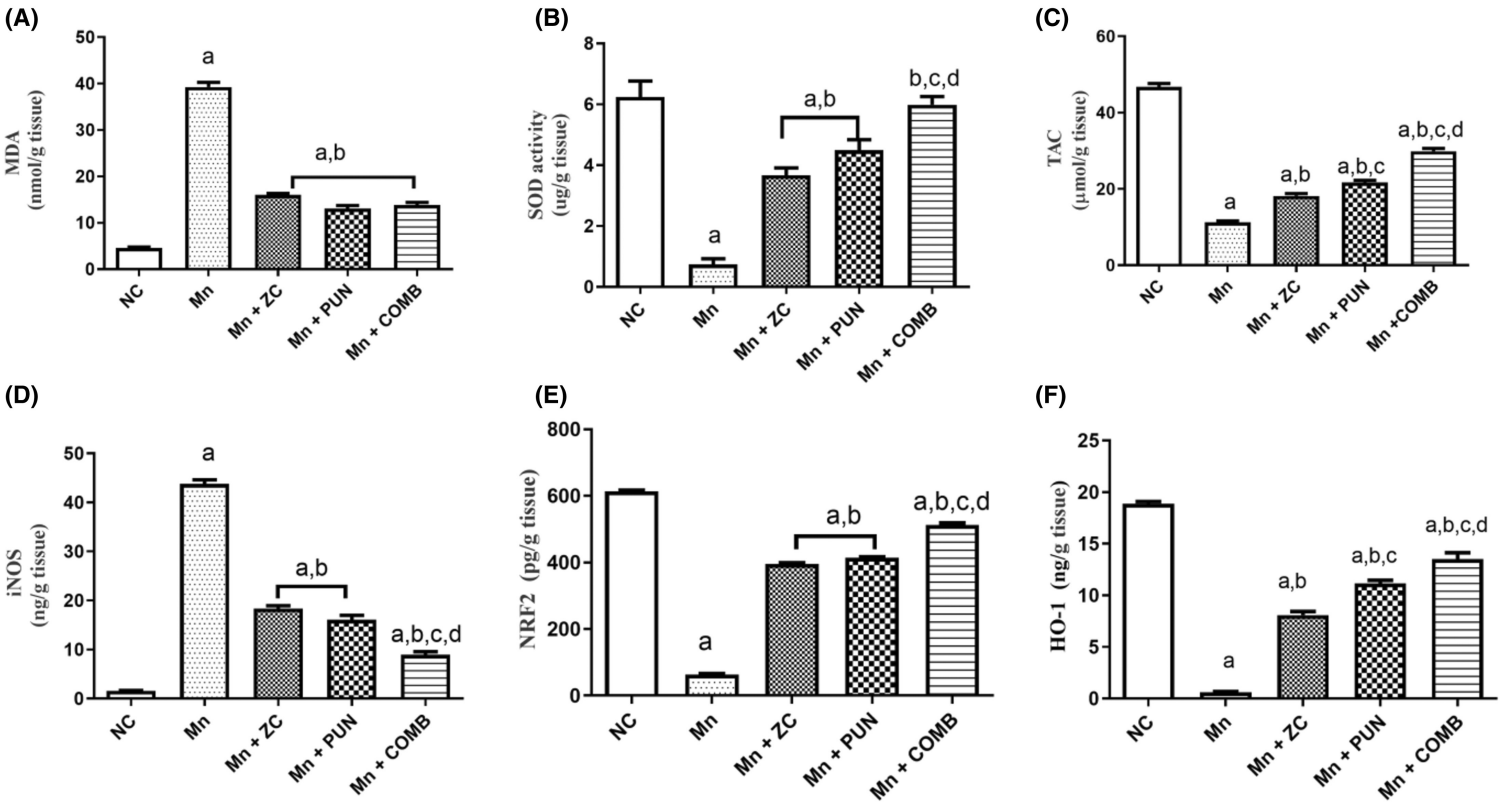
Effect of micronized zeolite clinoptilolite and punicalagin, either alone or in combination, on brain lipid peroxidation, (A) MDA levels and the levels of the oxidative stress markers (B) SOD, (C) TAC, (D) iNOS, (E) Nrf2, and (F) HO‐1 in MnCl_2_‐induced PD. NC: Normal control rats, Mn, MnCl_2_, control group, Rats treated daily with MnCl_2_ for 5 weeks to develop PD, Mn + ZC, Rats that received MnCl_2_ + Zeolite clinoptilolite nanoparticles, Mn + PUN: Rats that received MnCl_2_ + punicalagin, Mn + COMB, Rats that received MnCl2 + drug combinations (ZC and PUN), PD, Parkinson's disease, ZC, Zeolite clinoptilolite, PUN, Punicalagin, COMB, Combination of ZC and MnCl_2_, Nrf2, Nuclear factor erythroid 2‐related factor, TAC, Total antioxidant capacity, MDA, Malondialdehyde, SOD, Superoxide dismutase, iNOS, Inducible nitric oxide synthase, and HO‐1, Heme‐oxygenase‐1. The data are presented as the means ± SEMs, *n* = 6/group; ^a^significant difference from NC; ^b^significant difference from Mn. For parametric data, one‐way ANOVA was performed followed by Tukey's multiple comparisons test to assess group variation, with a significance level set at *p* < 0.05, while the Kruskal–Wallis test was used for nonparametric data, followed by Dunn's post hoc multiple comparison tests.

Mn dramatically reduced the brain's total antioxidant capacity (TAC) (Figure 2A), an oxidative stress biomarker, by approximately 88.8% and increased the brain (MDA) (Figure 2C), lipid peroxidation biomarker, and iNOS (Figure 2D) levels (approximately 8.7‐ and 28.1‐fold, respectively), relative to those in the normal control. In contrast to those of Mn‐treated rats, rats supplemented with ZC, PUN, or their combination displayed significantly greater brain TAC (55.5%, 60%, and 71.4%, respectively), significantly lower MDA content (approximately 2.4, 3.0, and 2.8%, respectively), and significantly greater iNOS content (2.3‐, 2.7‐, and 4.8‐fold, respectively), which were comparable to those of the PD group. The combination therapy had a tremendous effect on oxidative stress parameters, particularly TAC and iNOS, while there were no noticeable differences in the decreasing effects on MDA among the three treatment groups. Moreover, the MnCl2‐injected group exhibited substantial decreases in Nrf2 (Figure 2E) and HO‐1 (Figure 2F) levels and SOD activity (Figure 2B) of more than 89%, 95%, and 83.3%, respectively, compared with those in the control group. Treatment with ZC, PUN, or their combination markedly restored the levels of Nrf2, HO‐1, and SOD to nearly normal levels compared to those in the Mn‐treated group. Compared with monotherapy, combined therapy had the greatest influence on Nrf2 and HO‐1 levels and on SOD activity (Figure 2).

Therefore, the protective effect of ZC was comparable to that of PUN, as ZC has a protective effect against Mn‐induced oxidative stress by reducing lipid peroxidation and Nrf2 biomarker levels. The combination of ZC and PUN also had a synergistic effect on protection against the reductions in all measured oxidative stress and lipid peroxidation biomarker levels. There was no apparent difference in the effects of ZC, PUN, or their combination on the control group or the typical control group. These findings revealed that monotherapy and combination treatment could reverse oxidative stress, lipid peroxidation, and cytoprotective marker levels to almost normal levels.

### 3.4 Effect of ZC or PUN alone or in combination on brain proinflammatory and inflammatory biomarker levels in MnCl_2_ ‐induced PD

**FIGURE 3:**
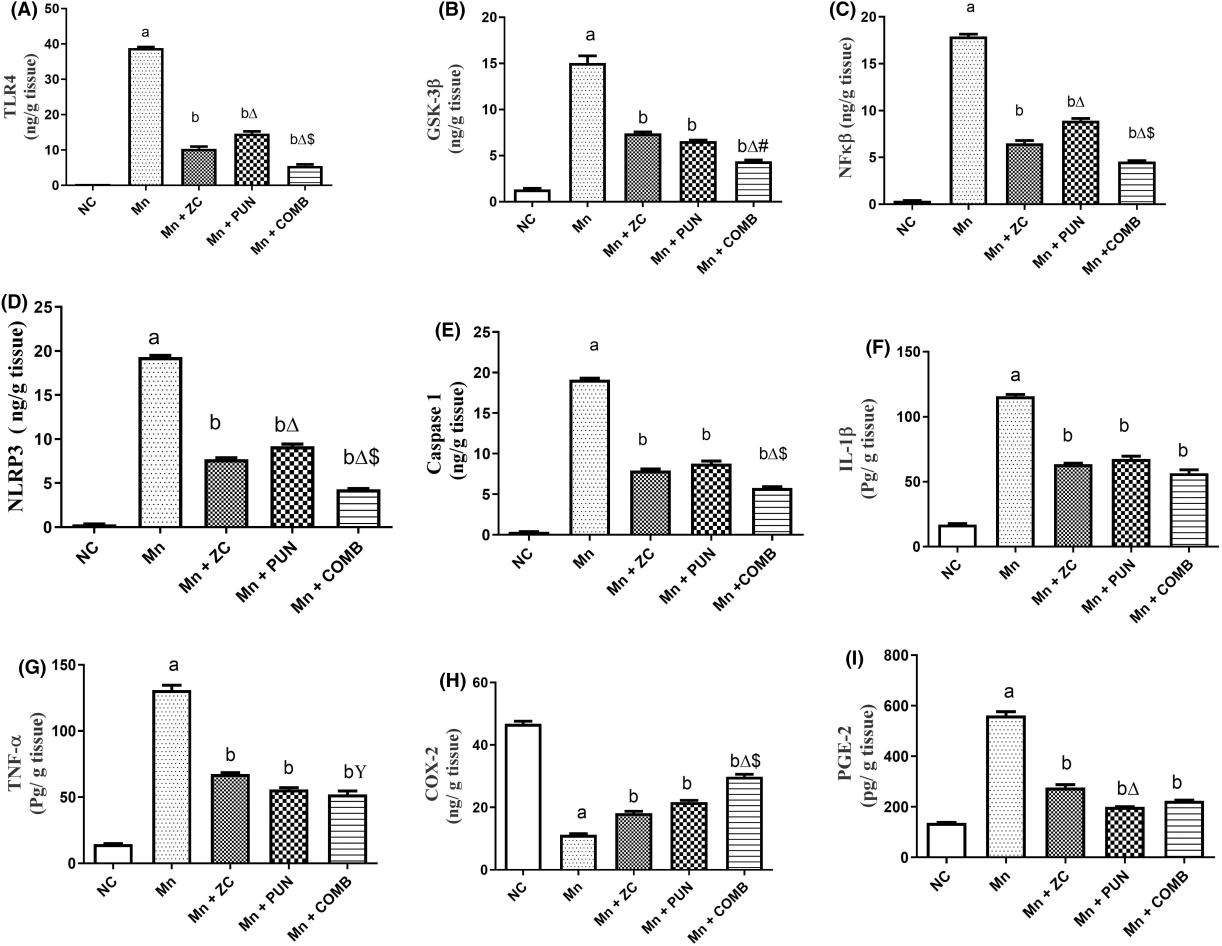
Effect of micronized zeolite clinoptilolite and punicalagin either alone or in combination on brain proinflammatory, inflammatory, and inflammasome activation biomarkers in MnCl_2_‐induced PD. NC, Normal control rats fed a rodent chow diet; Mn, MnCl_2_, control group, Rats were treated daily with MnCl_2_ for 5 weeks to induce PD; Mn + ZC, Rats received MnCl_2_ + micronized zeolite clinoptilolite; Mn + PUN, Rats received MnCl_2_ + punicalagin; Mn + COMB, Rats received MnCl_2_+ drug combinations (ZC and PUN); PD, Parkinson's disease; ZC: Zeolite clinoptilolite; COMB, Combination of ZC and MnCl_2_; PUN, Punicalagin; TLR‐4, Toll‐like receptor 4; GSK‐3β, Brain‐derived neurotrophic factor; IL‐Iβ, Interleukin‐1β; TNF‐α, Tumor necrosis factor alpha; COX‐2, Cyclo‐oxygenase 2; NLRP3, NLR family pyrin domain containing 3; NF‐kB, Nuclear factor kappa kβ; PGE‐2, Prostaglandin E2; Caspase‐1, Cysteine‐aspartic protease‐1. The data are presented as the means ± SEMs, *n* = 6/group; ^a^significant difference from NC; ^b^significant difference from Mn. For parametric data, one‐way ANOVA was performed followed by Tukey's multiple comparisons test to assess group variation, with a significance level set at *p* < 0.05, while the Kruskal–Wallis test was used for nonparametric data, followed by Dunn's post hoc multiple comparison tests.

Compared with those in the controls, the expression of genes regulating the inflammatory response, TLR4 (Figure 3A), NF‐kB (Figure 3B), and GSK‐3β (Figure 3C), was markedly increased in the MnCl_2_‐injected animals. ZC, PUN, and their combinations significantly decreased the levels of these parameters relative to those in the MnCl_2_ group by approximately 73.4%, 62.3%, and 85.8%, respectively, for TLR4; 63.6%, 50.2%, and 74.8%, respectively, for NF‐kB; and 73.4%, 50.8%, 56.3%, and 71.3%, respectively, for GSK‐3. Therefore, PUN had a greater protective effect against Mn‐induced increases in the levels of TLR4 and NF‐kB than did micronized ZC, while each treatment had the same effect on the increase in the level of GSK‐3. The combination of ZC and PUN also had a synergistic impact on the increase in the levels of all the assessed neuroinflammatory biomarkers TLR4, NF‐kB, and GSK‐3B. In addition, combination therapy had a significant influence on TLR4, NF‐kB, and GSK‐3B compared to those in the monotherapy groups (Figure 3).

MnCl_2_ markedly increased the neuro‐inflammatory brain levels of IL‐1β (Figure 3F), TNF‐α (Figure 3A), PGE‐2 (Figure 3B), and COX‐2 (Figure 3C) by approximately 110%, 92.8%, 72.4%, and 317.5%, respectively, compared with those in the NC group. Conversely, pretreatment with ZC, PUN, or their combination significantly reduced brain IL‐1β levels by 45.2%, 41.7%, and 51.2%, respectively; TNF‐α levels by approximately 52.0%, 56.0%, and 60.0%, respectively; PGE‐2 levels by approximately 56.8%, 65.5%, and 63.7%, respectively; and COX‐2 levels by approximately 36.1%, 53.6%, and 61.1%, respectively, similar to those in the Mn‐induced PD group. The combination therapy has a superior effect in halting the increase in the level of COX‐2 caused by exposure to MnCl_2_. Therefore, the protective effect of ZC on TNF‐α, PGE‐2, and COX‐2 was comparable to that of PUN, while for IL‐1β, PUN had a greater effect. These findings revealed the protective effect of these compounds against Mn‐induced elevations in inflammatory biomarker levels. In addition, the combination of ZC and PUN had a synergistic effect on all measured inflammatory biomarker levels.

Compared with control rats, rats given MnCl_2_ displayed nearly maximal increases in the levels of the inflammasome NLRP3 (Figure 3D) and Caspase‐1 (Figure 3E) (20 times). ZC, PUN, and their combination significantly decreased the level of NLRP3 by 60.1%, 52.8%, and 78.2%, respectively, and the level of Caspase‐1 by 58.6%, 54.4%, and 70%, respectively, in the MnCl_2_‐injected group. As a result, ZC exhibited similar protection to PUN, which has been shown to protect against Mn‐induced elevations in the activity of the inflammasome activation biomarker caspase‐1 and to return their levels to normal levels. The combination therapy had the greatest improvement effect on the NLRP3 and Caspase‐1 levels. ZC and PUN together had synergistic protective effects on neurodegeneration biomarkers.

### 3.5 Effect of ZC or PUN alone or in combination on brain ER stress, autophagy, apoptosis, and neurodegenerative biomarker levels in MnCl_2_ ‐induced PD

**FIGURE 4:**
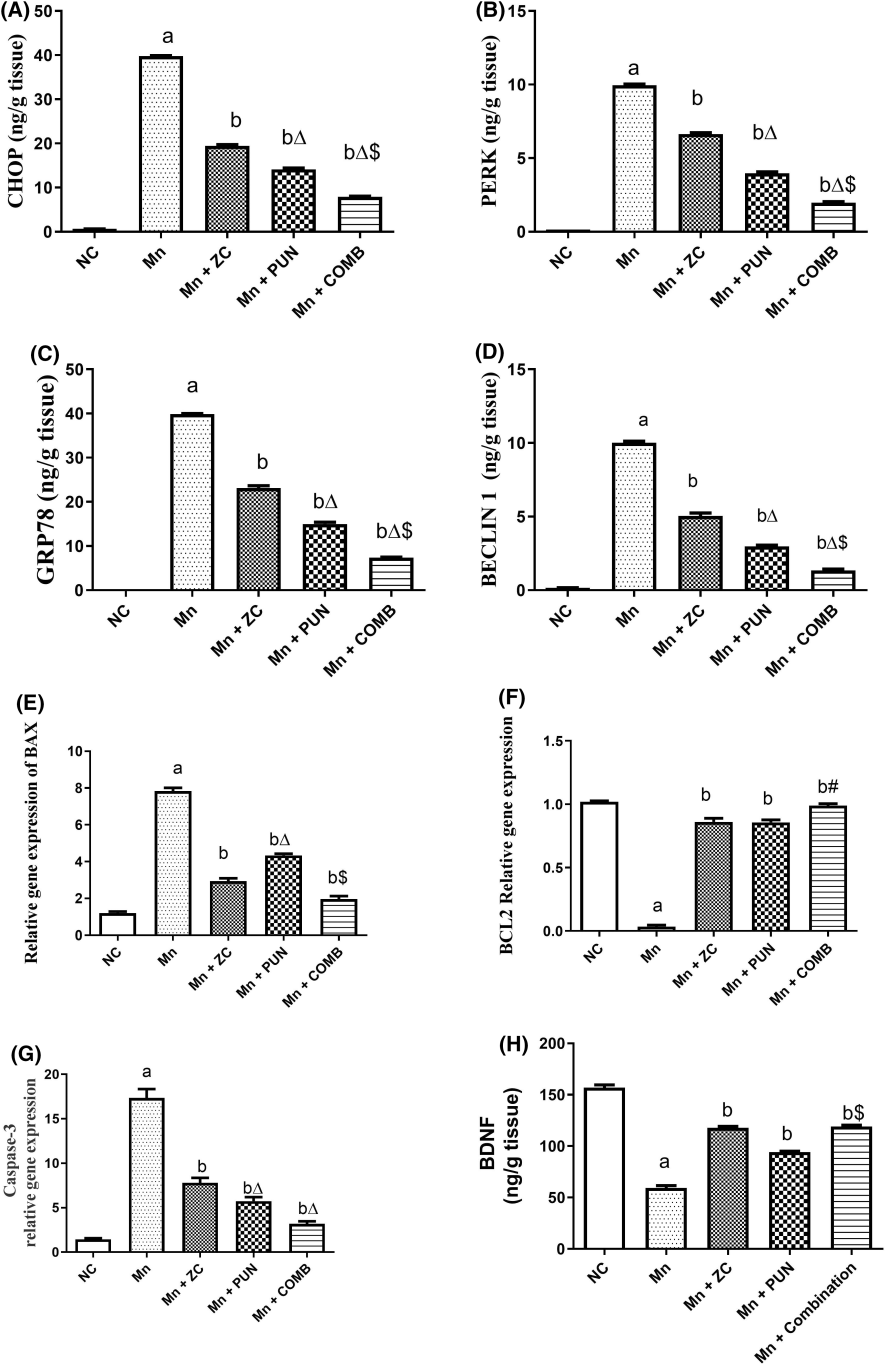
Effect of micronized zeolite clinoptilolite and punicalagin either alone or in combination on ER stress, autophagy, apoptosis, and neurodegenerative biomarkers in MnCl_2_‐induced PD. NC, Normal control rats fed a rodent chow diet; Mn, MnCl_2_, control group, Rats were injected daily with MnCl_2_ for 5 weeks to progress PD; Mn + ZC, Rats received MnCl_2_ + zeolite‐micronized clinoptilolite; Mn + PUN, Rats received MnCl_2_ + punicalagin; Mn + COMB, Rats received MnCl_2_ + drug combinations (ZC and PUN); PD, Parkinson's disease; ZC, zeolite clinoptilolite; PUN, Punicalagin; COMB, Combination of ZC and MnCl_2_; GRP78, Glucose Regulated Protein 78; p‐PERK, Phospho‐protein kinase RNA‐like ER kinase; Beclin‐1, Bcl‐2‐homology (BH)‐3 domain; Bcl‐2, B‐cell lymphoma‐2; Bax, Bcl‐2‐associated X apoptosis regulator C/EBP‐homologous protein; BDNF, Brain‐derived neurotrophic factor; CHOP, C/EBP homologous protein. Data are represented as the mean ± SEM, *n* = 6/group; ^a^significant difference from NC, ^b^significant difference from Mn. For parametric data, one‐way ANOVA was performed followed by Tukey's multiple comparisons test to assess group variation, with a significance level set at *p* < 0.05, while the Kruskal–Wallis test was used for nonparametric data, followed by Dunn's post hoc multiple comparison tests.

Figure 4 shows the estimated ER stress and autophagy parameters. Assessment of PERK (Figure 4A), CHOP (Figure 4B), and GRP78 (Figure 4C) revealed that the PD‐like conditions caused by MnCl_2_ in rats considerably altered the levels of ER stress indicators (Figure 4D). MnCl_2_ reduced the level of the autophagy marker BECLIN‐1 compared with that in the healthy control group (Figure 4D). Compared with the corresponding untreated animals, the animals treated with ZC, PUN, and their mixture exhibited significant improvements in these parameters. In addition, combination therapy has the strongest impact on preventing ER stress and enhancing autophagy, which works together to reverse the harmful effects of MnCl_2_‐induced PD. Therefore, ZC and PUN together provided a synergistic protective effect against elevated levels of all evaluated cognitive impairments and neurodegeneration biomarkers.

Moreover, animals given MnCl_2_ for the induction of PD‐like syndrome displayed an abnormally strong proapoptotic effect on neural cells. The overexpression of the apoptotic regulator *Bax* (Figure 4E) (8.3‐fold increase) and downregulation of *Bcl‐2* (anti‐apoptotic) (Figure 4F) (92% decrease) confirmed this effect. Notably, compared with no Mn treatment, monotherapy, or combined therapy significantly inhibited apoptosis by reversing the imbalance in apoptotic markers. The combination therapy had the greatest inhibitory effect on apoptosis.

The protective effect of PUN, which has been confirmed to have a protective effect against Mn‐induced decreases in the activity of the antiapoptotic biomarker *Bcl‐2*, was thus comparable to that of ZC. Interestingly, the combination of ZC and PUN provided a potent protective effect against increased levels of all the evaluated biomarkers of neurodegenerative and cognitive impairment. There was no discernible difference between the effects of ZC, PUN, or their combination and those of the normal control group.

In addition, MnCl_2_ markedly upregulated *caspase‐3* expression in brain tissue (8.5‐fold) relative to that in control rats. ZC, PUN, and the coadministration of PUN with ZC downregulated caspase‐3 mRNA expression (Figure 3G) (2.4‐, 3.4‐, and 5.7‐fold, respectively), compared to that in the Mn‐induced PD group. Notably, the combination group had no advantage in terms of apoptosis markers compared to the other monotherapy groups.

Compared with those in the control group, the BDNF activity in the brains of the rats in the MnCl_2_ group significantly decreased (Figure 3H) by approximately 46.4%. Conversely, compared with Mn‐induced PD, pretreatment with ZC, PUN, or their combination significantly reversed the reductions in BDNF levels by approximately 98.8%, 58.5%, and 100%, respectively. Conversely, pretreatment with ZC, PUN, or their combination significantly reduced BDNF levels by approximately 98.8%, 58.5%, and 100%, respectively, compared to those in the Mn‐induced PD group. The combination effect on BDNF activity in the brain was more notable in the PUN‐treated group than in the MnCl2‐treated group. The combination effect on BDNF activity in the brain was more prominent in the PUN‐treated group than in the MnCl_2_‐treated group.

### 3.6 Effect of ZC or PUN alone or in combination on histopathological alterations in brain tissue histopathology in MnCl_2_ ‐induced PD patients

**FIGURE 5:**
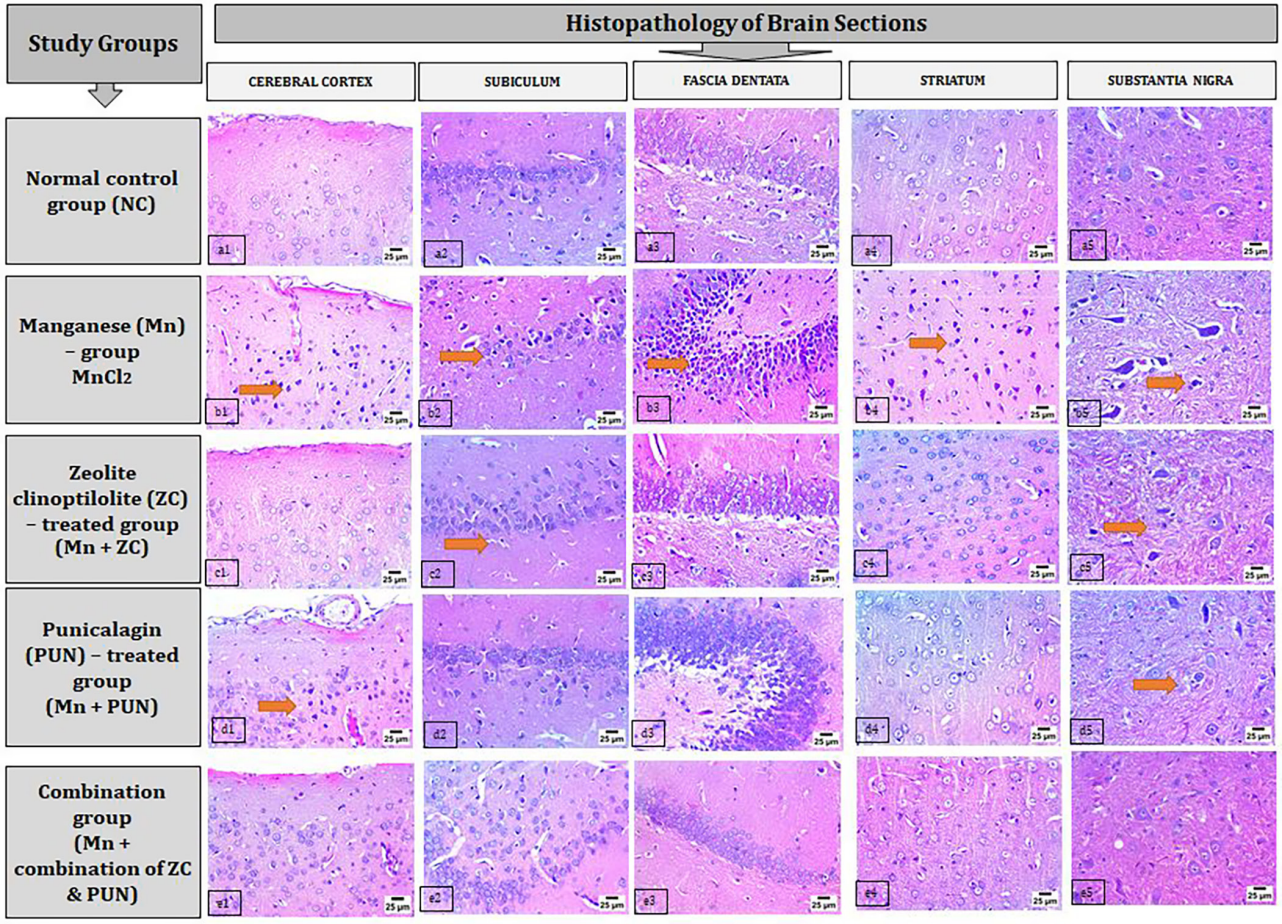
Photomicrographs of brain sections (cerebral cortex, subiculum, and fascia dentata in the hippocampus, striatum, and substantia nigra) (magnification 40×). Where (a1, a2, a3, a4, a5) are the normal control group (NC); (b1, b2, b3, b4, b5) are the manganese (Mn) group; (c1, c2, c3, c4, c5) are the zeolite clinoptilolite (ZC) treatment group (Mn + ZC); (d1, d2, d3, d4, d5) are the punicalagin (PUN) treatment group (Mn + PUN); and (e1, e2, e3 e4, e5) are the combination group (Mn + combination of ZC and PUN). In brain sections, the orange arrow indicates nuclear pyknosis and degeneration.

In the normal control group (NC), there was no histopathological alteration, and the normal histological structure of the neurons was recorded in all regions of the brain (cerebral cortex, subiculum, and fascia dentata in the hippocampus, striatum, and substantia nigra) (Figure 5a1, a2, a3, a4, a5). In the Mn‐treated group, severe nuclear pyknosis and degeneration were detected in the neurons of the cerebral cortex (Figure 5b1) and the subiculum of the hippocampus (Figure 5b2). Nuclear pyknosis was also detected in some neurons of the fascia dentate of the hippocampus (Figure 5b3). In addition, the striatum of the brain showed the formation of multiple nuclear pyknosis and degeneration in some neurons (Figure 5b4). Additionally, severe nuclear pyknosis and degeneration were observed in neurons in the substantia nigra (Figure 5b5). In the Mn + ZC‐treated group, neurons in the cerebral cortex exhibited an intact histological structure (Inset c1), whereas a few neurons in the subiculum of the hippocampus showed signs of degeneration and nuclear pyknosis (Figure 5c2). There were no changes observed in the neurons of the fascia dentate within the hippocampus (Figure 5c3) or in the striatum (Figure 5c4). However, degeneration was noted in certain neurons of the substantia nigra (as illustrated in Figure 5c5). In the PUN‐treated group (Mn + PUN), nuclear pyknosis and degeneration were detected in all the neurons of the cerebral cortex (Figure 5d1). There were no alterations in the neurons of the subiculum (Figure 5d2), fascia dentate of the hippocampus (Figure 5d3), or striatum (Figure 5d4). Additionally, nuclear pyknosis was detected in neurons in the substantia nigra of the brain (Figure 5d4). In the combination group (Mn + ZC + PUN), there were no histopathological alterations in the cerebral cortex (Figure 5e1), subiculum (Figure 5e2), fascia dentata of the hippocampus (Figure 5e3), striatum (Figure 5e4), or substantia nigra (Figure 5e5).

## 4 DISCUSSION

The accumulation of MnCl_2_ in the brain gives rise to manganism, a condition associated with behavioral and intellectual impairments reminiscent of Parkinsonism.^5^, ^6^ Multiple intricate mechanisms, including oxidative stress, ER stress, neuroinflammation, apoptosis, and disruption of neurotransmitter synthesis and metabolism, contribute to the cellular response leading to manganism.^12^ However, natural compounds have emerged as potential modulators of these cellular stress reactions, offering promising avenues for developing pharmacological interventions for various neurological disorders, including neurodegenerative illnesses.^41^ Thus, natural products have become a captivating resource for identifying potential therapeutic candidates.

Among these natural compounds, PUN, an ellagitannin abundantly found in pomegranate fruit, has garnered significant attention due to its myriad of health benefits.^35^, ^36^ Notably, it has remarkable therapeutic potential in the management of various disorders, including neurological disorders.^37^, ^38^ Researchers attribute its therapeutic effects to its ability to repress oxidative stress, inflammatory cascades, and apoptosis.^39^, ^61^, ^62^ Subkorn et al. showed that PUN is distributed to brain tissues through intestinal metabolism via the microbiota.^61^ Therefore, after oral administration, it is transferred to brain tissues through the gut‐microbiota‐brain axis. Therefore, we used an oral route of administration for PUN to study its neuroprotective effects.

ZC is a versatile naturally occurring mineral with a remarkable ability to capture a wide range of ions and molecules, including heavy metals such as Mn.^41^ The unique properties of ZC make it a valuable material for various industrial and biomedical applications and therapeutic approaches, including chelation, detoxification, anti‐inflammatory, immune boosting, anticarcinogenic, and antioxidative activities.^41^, ^44^ Despite its widespread use, the precise mechanisms underlying the therapeutic potential of ZC, particularly its neurological effects, remain elusive.^41^ The distinctive structure of ZC and its ability to absorb and exchange ions enable interactions with various ionic groups and molecules, including heavy metals, ammonia, and nitrates.^41^ Additionally, ZC acts as an antioxidant by neutralizing free radicals and reducing the synthesis of reactive oxygen species (ROS). Moreover, it can activate antioxidant enzymes by providing metal ions from within its structure as cofactors. Encouraging results from animal studies suggest that the detoxifying properties of micronized ZC at the intestinal and systemic levels may be responsible for its antioxidant effect. Furthermore, the pores of micronized ZC can interact with various organic molecules and ions, altering the calcium ion concentration and affecting Ca‐dependent molecular signals.^41^ Kraljević et al. showed that micronized ZC is transferred to brain tissues through the CSF after being metabolized in the intestinal microbiota.^50^ Therefore, we administered ZC orally to brain tissues through the gut–microbiota–brain axis. Therefore, we used an oral route of ZC administration to study its neuroprotective effects.

With an in‐depth understanding of manganese‐induced PD, our research aimed to investigate the neuroprotective effects of PUN, micronized ZC, and their combination in a rat model of PD induced by MnCl_2_ exposure. Our focus was on exploring the intricate mechanisms through which these agents improve PD symptoms, with a particular emphasis on autophagy, the PERK/CHOP/Bcl‐2 pathway, and the Nrf2/HO‐1 cytoprotective signaling pathway in addition to the TLR4/NF‐kβ/NLRP3 pathway. By delving into these pathways, we aspired to shed light on potential therapeutic strategies for countering the detrimental effects of manganese‐induced PD, thus paving the way for enhanced neuroprotection.

In our grid and bar tests, our analysis revealed that rats exposed to MnCl_2_ displayed a cataleptic reaction, indicating the emergence of bradykinesia and rigidity in the animals. Moreover, motor dysfunction was observed in the OFT. Long‐term overexposure to MnCl_2_ in the brain led to severe alterations in histopathology throughout all brain regions, particularly impacting the basal ganglia, including the globus pallidus projection, resulting in significant dopamine neuronal cell loss. This disruption in basal ganglia neurotransmitters contributed to the observed behavioral alterations.^63^ Consistent with previous findings,^10^, ^64^ rats exposed to MnCl_2_ exhibited altered histological findings in the striatum, characterized by the presence of nuclear pyknosis in certain neurons, compared to the normal striatum and tissue of the nonparkinsonism group. These histological changes in the brain can also affect brain dopamine metabolism, as well as glutamatergic and GABAergic activity.^65^

Our research further indicated a substantial reduction in brain monoamine levels, including dopamine (DA), norepinephrine (NE), and serotonin (5‐HT), in the hippocampus, as well as elevated AChE activity, consistent with earlier studies.^52^, ^66^, ^67^ Excessive Mn exposure was found to induce prominent glutamate excitotoxicity, accompanied by a considerable decrease in inhibitory GABA, the main inhibitory neurotransmitter.^68^ An increase in glutamate levels because of exposure to manganese induces the synthesis of ROS, which could prohibit glutamate transporters from clearing extracellular glutamate.^69^ These results provide the groundwork for linking oxidative stress with the disturbance of neurotransmission in the basal ganglia.

However, ZC, PUN, and their combination treatment reduced MnCl_2_‐induced catalepsy and improved locomotor impairment, which led to the recovery of brain monoamine (DA, NE, and 5‐HT) levels and a decrease in AChE activity. Therefore, the present study confirmed the neuroprotective ability of ZC, PUN, and their combination since they prevented MnCl_2_ distortion in the striatum, resulting in an intact histological structure, decreased degeneration, pyknosis, and preserved brain tissue. Previous behavioral research supported our findings that food enriched with ZC enhanced the ability of the animals to resist and react to environmental stress.^50^ In addition, the micronized ZC might have a positive effect on serotonergic receptors in mammary carcinoma‐bearing animals because of the impacts of ZC on electrolyte hemostasis and its neuro‐immunomodulatory actions.^51^ PUN minimized motor function deficits following I/R in rats, revealing its therapeutic potential.^49^ Furthermore, after receiving pomegranate extract, the levels of DA and its metabolite DOPAC were restored.^35^, ^36^ The same extract also effectively reversed the cognitive deficits induced by scopolamine in rats because it dramatically reduced AChE expression in the hippocampus.^57^ Furthermore, our data confirmed that the investigational medicines reduced the increase in excitatory glutamate and restored glutamate/GABA equilibrium in the basal ganglia.

According to the current study, brain tissue showed increased levels of MDA and iNOS, which are oxidative stress biomarkers, after MnCl2 treatment, and TAC decreased. These results agree with previous data showing that overexposure to MnCl_2_ induces oxidative stress and increases free radical activity, leading to the oxidation of DNA, lipids, and proteins in brain tissue and thereby elevating oxidative stress biomarkers.^8^, ^70^ All the rats treated with the selected medications and their mixtures exhibited antioxidant effects via the regulation of the stress markers MDA, iNOS, and TAC. Notably, ZC was found to completely inhibit nitric oxide production in vivo in metastatic melanoma.^59^ Furthermore, ZC therapy reduced lipid peroxidation and MDA levels and enhanced antioxidant capacity in smokers.^71^ A recent report highlighted the neuroprotective impact of PUN through the attenuation of nitrite and MDA levels.^49^ Moreover, PUN has been associated with reducing the risk and progression of diabetic liver damage through its ability to mitigate oxidative stress.^62^

A crucial regulator of the adaptive survival response to oxidative stress, Nrf2, is believed to play a significant role in upregulating cytoprotective enzymes.^72^ This transcription factor binds to antioxidant response elements, thereby controlling the expression of essential antioxidant markers such as SOD, CAT, GSH, and HO‐1.^72^ The Nrf2/HO‐1 pathway is a crucial element in protecting nerve cells against oxidative damage induced by manganese chloride.^12^ In our study, we observed that overexposure to Mn significantly suppressed the brain levels of Nrf2, HO‐1, and SOD in experimental animals. These findings corroborated previous research showing altered Nrf2 expression and activity in nigral dopaminergic neurons of PD patients.^12^ However, the administration of ZC, PUN, or a combination of the two reversed the changes in Nrf2 and HO‐1 levels. PUN demonstrated a protective effect by increasing HO‐1 expression through a mechanism that involves Nrf2 translocation and enhancing the antioxidant defense system with increased CAT and SOD levels.^39^, ^73^, ^74^ These findings were consistent with earlier studies in which ZC treatment after plaque formation in mice reduced ROS‐induced cell death in SH‐SY5Y neuronal‐like cells and boosted SOD activity.^75^ Although the protective impact of ZC on the Nrf2/HO‐1 pathway has not been specifically studied, these results highlight the potential of targeting Nrf2/HO‐1 as a therapeutic strategy for mitigating oxidative stress and acting as a cytoprotective agent in manganism and possibly PD.

On the other hand, oxidative stress serves as a primary instigator of inflammation and apoptosis, both of which play pivotal roles in brain injury.^76^ MnCl_2_ exposure was found to increase the levels of proinflammatory factors, such as NF‐κB and TLR4, and inflammatory cytokines, such as IL‐1β, PGE‐2, and TNF‐α, thus confirming the activation of proinflammatory pathways. Additionally, Mn is known to induce neuroinflammation by modulating pathways involving TLR4, NF‐κB, COX‐2, and NLRP3/Caspase‐1, as well as disrupting mitochondrial dynamics.^6^, ^10^, ^12^, ^19^, ^77^, ^78^, ^79^, ^80^ Furthermore, our present research demonstrated the suppressive effects of ZC, PUN, and/or their combination on Mn‐induced neurotoxicity, effectively reducing the levels of NF‐κB, TLR4, IL‐1β, PGE‐2, and TNF‐α in the brain. Previous studies have reported that ZC significantly reduces inflammation in animals with induced esophagitis.^81^ Additionally, PUN has been shown to be a potent inhibitor of TLR4 activation and NF‐κB, as well as to downregulate NLRP3 expression induced by LPS, leading to a substantial decrease in proinflammatory cytokine production during oxidative stress.^39^, ^40^, ^52^, ^82^, ^83^, ^84^

Moreover, in response to cellular injury, the innate immune system's NLRP3 component triggers caspase‐1 activation and the release of the proinflammatory cytokine IL‐1β, which can be induced by Mn.^85^, ^86^, ^87^ IL‐1β plays a crucial role in the induction of mitochondrial apoptosis.^88^ These findings suggest that drugs that inhibit the NLRP3 inflammasome may be suitable for both curative and preventive treatment of neurological diseases. In our current study, we found that ZC, PUN, and their combination effectively counteracted MnCl_2_‐induced neurotoxicity by inhibiting NLRP3 inflammasome‐dependent pyroptosis. Earlier research has shown that PUN therapy for diabetic nephropathy may reduce the production of pyroptosis‐related proteins, including caspase‐1, NLRP3, and the inflammatory cytokine IL‐1β, in renal tissue.^83^ The protective effects of PUN and ZC rely heavily on their ability to inhibit the inflammatory response caused by pyroptosis.

Moreover, in the process of PD development, the multifunctional kinase glycogen synthase kinase‐3 (GSK‐3) plays a role as a proinflammatory and proapoptotic kinase.^89^ In our study, we discovered that Mn intoxication could enhance GSK‐3β activity in the rat brain, which is consistent with the findings of an earlier investigation.^12^, ^90^ GSK‐3 blockade can reduce oxidative injury in various types of neurons, making GSK‐3 deactivation a significant mediator of the reduction in neuronal oxidative stress.^91^, ^92^ Our present findings support this view, as we observed that ZC, PUN, and their combination therapy effectively suppressed brain GSK‐3β levels. However, the specific protective impact of ZC alone or in combination with PUN on GSK‐3β levels has not been previously assessed. Further research in this area could shed more light on the potential neuroprotective mechanisms of these agents against GSK‐3β‐mediated oxidative stress in PD.

Notably, BDNF levels in serum or plasma could serve as a biomarker for neurodegenerative alterations.^93^, ^94^ In addition to assisting in the solidification of long‐term memory, BDNF also plays a crucial role in the differentiation and survival of CNS neurons.^95^ Additionally, it exerts a potent protective effect against neurodegeneration by activating the PI3K/AKT pathway, leading to the inactivation of GSK3β, which effectively inhibits apoptosis.^96^ Our results showed that manganese administration was inversely correlated with BDNF levels, which is consistent with earlier research.^18^ However, ZC, PUN, and their combination had a significant positive effect on the BDNF level. To the best of the author's knowledge, this is the first study to measure the protective effect of ZC on BDNF levels. Previous studies have shown that pomegranate extract mixed with different polyphenols restored BDNF levels in humans.^97^

Growing evidence suggests a connection between ER stress, oxidative stress, and redox signaling agents.^98^ Neurodegenerative diseases and ischemia/reperfusion injury have also been linked to ER stress.^99^, ^100^, ^101^, ^102^, ^103^, ^104^ Therefore, based on our findings, it is evident that MnCl2 toxicity activates ER stress, as evidenced by a significant increase in the levels of ER stress biomarkers such as GRP78, CHOP, and phosphorylated PERK. Prolonged exposure to extreme ER stress can lead to cell death through the activation of apoptotic pathways. In our current study, we observed that ZC, PUN, and their combination have the potential to reduce the levels of ER stress biomarkers, including GRP78, p‐PERK, and CHOP. Previous data also support our results, as they demonstrated that PUN can alleviate ER stress by suppressing p‐PERK and CHOP.^39^, ^105^, ^106^, ^107^ However, to the best of our knowledge, no previous study has assessed the effect of ZC on ER stress. This finding opens new avenues for investigating the potential role of ZC in modulating ER stress responses and its therapeutic implications in various diseases related to ER stress dysregulation, including neurodegenerative conditions.

ER stress can both stimulate and inhibit autophagy, and defective autophagy has been implicated in the development of PD.^108^ Autophagy plays a crucial role in clearing α‐synuclein in PD, and Beclin‐1 is commonly used as a biomarker to assess autophagic activity.^32^ Our results showed that overexposure to MnCl_2_ led to a reduction in the level of the autophagy marker Beclin‐1. This finding is consistent with an earlier study in PD patients, which also revealed decreased Beclin‐1 protein levels, indicating impaired clearance of α‐synuclein.^109^ To further investigate the impact of ZC, PUN, and their combination on autophagy, we examined the levels of Beclin‐1. Remarkably, we found that Beclin‐1 was significantly elevated, suggesting that ZC, PUN, and their combination may induce autophagy. These findings align with previous studies that demonstrated the protective effects of Beclin‐1 overexpression in activating autophagy and its role in various neurodegenerative diseases.^110^, ^111^ Overall, our study sheds light on the interplay between ER stress and autophagy in the context of MnCl_2_‐induced neurotoxicity and potential therapeutic interventions involving ZC and PUN. Further research in this area could provide valuable insights into novel approaches for managing PD and related neurodegenerative conditions.

Intriguingly, the interactions between ER stress and autophagy may collectively play a pivotal role in the regulation and initiation of apoptosis.^112^ The Bcl‐2 family of proteins, consisting of antiapoptotic Bcl‐2 and proapoptotic Bax, plays a crucial role in controlling apoptosis.^113^ Several lines of evidence have confirmed that Mn induces apoptosis in various cells^19^, ^77^, ^78^, ^114^ by triggering the caspase cascade,^115^ downregulating *Bcl‐2* expression, and upregulating *Bax* expression.^116^ Moreover, our study revealed that ZC deactivated the apoptotic pathway by restoring the level of Bcl‐2 and suppressing both caspase‐3 and Bax.^41^, ^75^, ^117^ Similarly, PUN was found to reduce apoptosis in the brain by upregulating Bcl‐2 expression and downregulating caspase‐3 and Bax.^18^, ^52^, ^63^ These results suggest that PUN and ZC alone or in combination may promote neuroprotection by regulating the expression of apoptotic proteins, indicating prevention of apoptosis and potential histological and neurotransmitter alterations and behavioral changes accompanying Mn‐induced PD.

## 5 CONCLUSIONS

In conclusion, as shown in the graphical abstract, our innovative research explored the intricate molecular pathways associated with manganese‐induced PD. Mn induces oxidative stress in brain tissues, leading to the elevation of oxidative stress biomarkers and downregulation of the cytoprotective Nrf2/HO‐1 pathway. This, in turn, triggers a cascade of events: increased GSK3β and decreased BDNF levels, activation of proinflammatory and inflammatory pathways such as TLR4/NF‐kβ/cytokines and inflammasomes, prolonged ER stress, impaired autophagy, and ultimately, apoptosis of neuronal cells. Consequently, these processes result in diminished neurotransmitter levels and lead to behavioral and histological alterations.

In our study, we also investigated the neuroprotective effects of PUN and micronized ZC, both individually and in combination, on Mn‐induced PD. These compounds effectively improved behavioral outcomes, histological structure, and biochemical markers in the brain. These compounds exhibited potent antioxidant, anti‐inflammatory, and neuroprotective effects, effectively countering the oxidative stress induced by Mn exposure. Furthermore, they modulated critical pathways, including the GSK3β/BNDF, cytoprotective Nrf2/HO‐1, NF‐κB/TLR4/NLRP3, and PERK/CHOP pathways, while promoting autophagy and inducing cell cytoprotection mechanisms, consequently restoring brain neurotransmitters to normal levels.

These exciting findings highlight the potential of micronized ZC and PUN as promising therapeutic agents for PD, paving the way for further research and offering hope for enhanced treatments.

### 5.1 The study limitations

1. The study's sample size was relatively small, potentially limiting the generalizability of the findings.
2. The use of animal models may not fully reflect the complexities of human PD pathology.
3. The short duration of treatment with micronized ZC and PUN warrants further investigation into long‐term effects.

### 5.2 The study strengths

1. This study comprehensively assessed behavioral, histological, and biochemical outcomes, providing a thorough understanding of the effects of micronized ZC and PUN.
2. This research revealed key molecular mechanisms underlying manganese‐induced PD, enhancing the understanding of disease progression.
3. The observed therapeutic potential of micronized ZC and PUN in mitigating oxidative stress and neuroinflammation underscores their promise for PD treatment.

### 5.3 Recommendation

1. We recommend conducting further studies on manganese toxicity to explore the effects of various manganese salts, including MgSO_4_. This will help broaden our understanding of the potential health impacts associated with different manganese compounds and their contributions to toxicity.
2. Furthermore, in future studies, we recommend focusing on Parkinson's disease in brain regions especially the basal ganglia, the substantia nigra, the striatum, and the midbrain. This targeted approach will provide deeper insights into the pathology and potential therapeutic interventions for Parkinsonism.
3. Additionally, given the antioxidant and anti‐inflammatory properties observed in micronized ZC and PUN, future investigations could explore Curcuma longa, or turmeric, as another herbal plant with similar mechanisms. Curcumin, an active compound, has neuroprotective effects on various neurodegenerative diseases, including Parkinson's disease. Further research into the potential of curcumin in manganese‐induced PD could elucidate its impact on oxidative stress, inflammation, and neuronal survival.

## AUTHOR CONTRIBUTIONS

Conceptualization, Karema Abu‐Elfotuh; Data curation, Karema Abu‐Elfotuh, Ahmed M.E. Hamdan, Amany B. Abdelrehim, Ayah M.H. Gowifel, and Alshaymaa Darwish; Formal analysis, Karema Abu‐Elfotuh, Ahmed M.E. Hamdan, Amany B. Abdelrehim, Ayah M.H. Gowifel, Alshaymaa Darwish, Ahmed M. Atwa, Magy R. Kozman, Ahmad Salahuddin, Amira M. Hamdan, Qutaiba A. Qasim, Aya H. Al‐Najjar, Shaimaa R Abdelmohsen, Heba Abdelnaser Aboelsoud, Amina M.A. Tolba, Ashwaq N. Abbas, Azza S. Khalil, Rana H. Abd El‐Rhman, Sara Nagdy Mahmoud Mousa, and Mazin A.A. Najm; Methodology, Karema Abu‐Elfotuh, Ahmed M.E. Hamdan, Amany B. Abdelrehim, Ayah M.H. Gowifel, and Alshaymaa Darwish; Supervision, Karema Abu‐Elfotuh; Validation, Karema Abu‐Elfotuh, Ayah M.H. Gowifel, and Alshaymaa Darwish; Writing—original draft, Amany B. Abdelrehim, Ayah M.H. Gowifel, and Alshaymaa Darwish; Writing—review and editing, Karema Abu‐Elfotuh, Ahmed M.E. Hamdan, Amany B. Abdelrehim, Ayah M.H. Gowifel, Alshaymaa Darwish, Ahmed M. Atwa, Magy R. Kozman, Qutaiba A. Qasim, Amira M. Hamdan, Aya H. Al‐Najjar, Shaimaa R Abdelmohsen, Heba Abdelnaser Aboelsoud, Amina M.A. Tolba, Ashwaq N. Abbas, Mazin A.A. Najm, Azza S. Khalil, Rana H. Abd El‐Rhman, Amira M. Negm, Ahmad Salahuddin, and Sara Nagdy Mahmoud Mousa. All authors have read and agreed to the final version of the manuscript.

## FUNDING INFORMATION

The research was not supported by external funds.

## CONFLICT OF INTEREST STATEMENT

The authors declare that the research was conducted in the absence of any commercial or financial relationships that could be construed as potential conflicts of interest.

## Supporting information


Data S1.

## Data Availability

The corresponding author will provide the datasets gathered during and/or analyzed during the present research upon reasonable request.
