# Supplementary material for: Neuroprotective effects of punicalagin and/or micronized zeolite clinoptilolite on manganese‐induced Parkinson's disease in a rat model: Involvement of multiple pathways
Source: CNS Neurosci Ther. 2024 Oct 7;30(10):e70008. doi: 10.1111/cns.70008 (PMC11457879; doi:10.1111/cns.70008)
Supplement: Supplementary file 1 — Data S1. [file CNS-30-e70008-s001.docx]

**Table (S1): The biochemical studies for control groups:**

| **Study group**  **Variable** | **Normal control (NC)**  Mean±SEM | **PUN treated group**  Mean±SEM | **ZC treated group**  Mean±SEM | **Combination treated group**  Mean±SEM |
| --- | --- | --- | --- | --- |
| **Dopamine**  (ng/g tissue) | 72.8±2.3 | 71±2.5 | 70±2.8 | 73±1.6 |
| **Norepinephrine** (nmol/g tissue) | 574.45±7.9 | 573.53±8.1 | 573.44±8.4 | 576.23±7.5 |
| **Serotonin**  (ng/g tissue) | 11.12±0.34 | 11.02±0.35 | 12±0.22 | 12.3±0.21 |
| **ACHE**  (ng/g tissue) | 23.7±1.27 | 22±1.8 | 22.6±1.7 | 23.5±1.12 |
| **Glutamate**  (ng/g tissue) | 11.2±0.44 | 10.7±0.6 | 11.9±0.22 | 11.5±0.23 |
| **GABA**  (ng/g tissue) | 7±0.73 | 6.9±0.72 | 7.2±0.53 | 7.1±0.63 |
| **MDA**  (nmol/g tissue) | 4.6±0.22 | 4.5±0.25 | 4.3±0.27 | 4.3±0.3 |
| **SOD**  (µgl/g tissue) | 6.24±0.5 | 6.11±0.53 | 6.2±0.54 | 6.13±0.43 |
| **TAC**  (µmol/g tissue) | 46.77±0.85 | 45.8±0.86 | 45.9±0.6 | 45.5±0.45 |
| **iNOS**  (ng/g tissue) | 1.55±0.08 | 1.6±0.07 | 1.45±0.09 | 1.45±0.07 |
| **Nrf2**  (pg/g tissue) | 612.9±2.9 | 614.9±1.9 | 615.9±2.6 | 613.9±2.8 |
| **HO-1**  (ng/g tissue) | 18.9±0.22 | 18.8±0.21 | 18.7±0.24 | 18.7±0.21 |
| **TLR4**  (ng/g tissue) | 0.4±0.01 | 0.35±0.05 | 0.4±0.03 | 0.3±0.09 |
| **GSK3β**  (ng/g tissue) | 1.33±0.13 | 1.32±0.14 | 1.33±0.12 | 1.32±0.2 |
| **NF-Kβ**  (ng/g tissue) | 0.39±0.017 | 0.38±0.018 | 0.37±0.019 | 0.37±0.017 |
| **NLRP3**  (ng/g tissue) | 0.32±0.023 | 0.31±0.024 | 0.31±0.022 | 0.3±0.022 |
| **Caspase -1**  (ng/g tissue) | 0.37±0.012 | 0.38±0.011 | 0.36±0.013 | 0.36±0.012 |
| **IL-1β**  (pg/g tissue) | 17±0.82 | 16.9±0.9 | 17±0.4 | 16.9±0.39 |
| **TNF-α**  (pg/g tissue) | 14.4±0.15 | 14.32±0.16 | 14.42±0.16 | 14.3±0.18 |
| **COX-2**  (ng/g tissue) | 11.2±0.45 | 11.3±0.4 | 11.3±0.35 | 11.1±0.5 |
| **PGE2**  (pg/g tissue) | 136.2±2.3 | 136.3±2.2 | 136.5±2 | 136 ±1.7 |
| **CHOP**  (ng/g tissue) | 0.62±0.004 | 0.61±0.003 | 0.59±0.005 | 0.6±0.003 |
| **PERK**  (ng/g tissue) | 0.16±0.001 | 0.17±0.001 | 0.159±0.002 | 0.163±0.001 |
| **GRP78**  (ng/g tissue) | 0.12±0.007 | 0.13±0.007 | 0.125±0.008 | 0.13±0.006 |
| **BECLIN-1**  (ng/g tissue) | 10.02±0.1 | 10.1±0.05 | 10.08±0.02 | 10.06±0.03 |
| **BAX**  (mRNA expression) | 1. 2±0.085 | 1. 1±0.09 | 1. 3±0.07 | 1. 1±0.075 |
| **BCL2**  (mRNA expression) | 1.0 2±0.008 | 1.0 3±0.007 | 1.0 4±0.006 | 1.0 3±0.008 |
| **Caspase 3**  (mRNA expression) | 1. 43±0.14 | 1. 4±0.15 | 1. 42±0.15 | 1. 4±0.13 |
| **BDNF**  (ng/g tissue) | 156.9±2.6 | 156.8±2.7 | 156.7±2.9 | 156.4±2.9 |

^a^ Significant deviations from the control group were determined with a p-value of less than 0.05, as assessed by a one-way ANOVA test. SEM refers to the standard error of the mean.


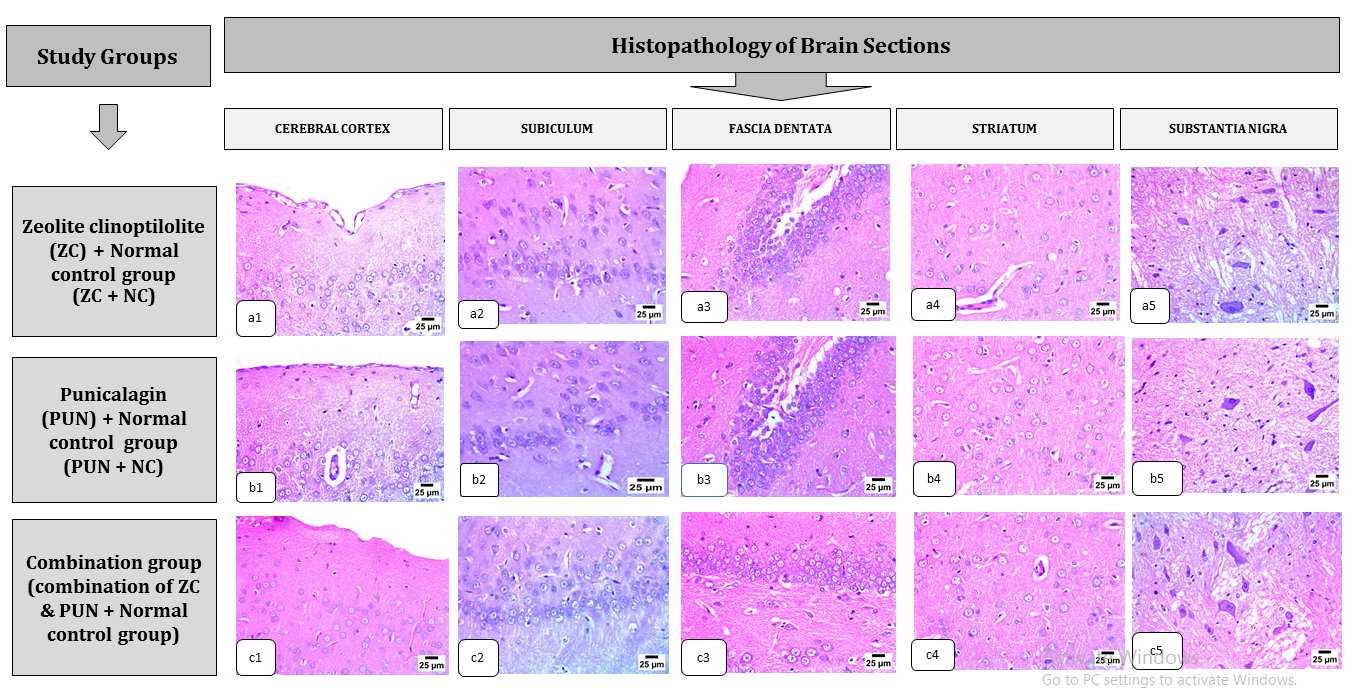


**Figure: (S1): Photomicrographs of brain sections (cerebral cortex, subiculum, and fascia dentata in the hippocampus, striatum, and substantia nigra) in control groups:** *(magnification 40X).* where (a1, a2, a3, a4, a5) are the zeolite clinoptilolite (ZC) + normal control (ZC + NC) group; (b1, b2, b3, b4, b5) are the punicalagin (PUN) + normal control (PUN + NC) group; (c1, c2, c3, c4, c5) are the combination group (combination of ZC & PUN + Normal control) group.
